# Zinc-finger nuclease mediated disruption of *Rag1* in the LEW/Ztm rat

**DOI:** 10.1186/1471-2172-13-60

**Published:** 2012-11-08

**Authors:** Nils-Holger Zschemisch, Silke Glage, Dirk Wedekind, Edward J Weinstein, Xiaoxia Cui, Martina Dorsch, Hans-Jürgen Hedrich

**Affiliations:** 1 Institute of Laboratory Animal Science, Hannover Medical School, Carl-Neuberg-Str.1, 30625, Hannover, Germany; 2 Sigma Advanced Genetic Engineering Labs, Sigma-Aldrich Corporation, 3050 Spruce Street, St. Louis, Missouri, 63103, USA

**Keywords:** *Rag1*, Zinc-finger nucleases, Rat, Lymphocytes, Natural killer cells, Hypoplastic thymus

## Abstract

**Background:**

Engineered zinc-finger nucleases (ZFN) represented an innovative method for the genome manipulation in vertebrates. ZFN introduced targeted DNA double strand breaks (DSB) and initiated non-homologous end joining (NHEJ) after pronuclear or cytoplasmatic microinjection into zygotes. Resulting frame shift mutations led to functional gene ablations in zebra fish, mice, pigs and also in laboratory rats. Therefore, we targeted the rat *Rag1* gene essential for the V(D)J recombination within the immunoglobulin production process and for the differentiation of mature B and T lymphocytes to generate an immunodeficient rat model in the LEW/Ztm strain.

**Results:**

After microinjection of *Rag1* specific ZFN mRNAs in 623 zygotes of inbred LEW/Ztm rats 59 offspring were born from which one carried a 4 bp deletion. This frame shift mutation led to a premature stop codon and a subsequently truncated *Rag1* protein confirmed by the loss of the full-length protein in Western Blot analysis. Truncation of the *Rag1* protein was characterized by the complete depletion of mature B cells. The remaining T cell population contained mature CD4^+^/CD3^+^/TCRαβ^+^ as well as CD8^+^/CD3^+^/TCRαβ^+^ positive lymphocytes accompanied by a compensatory increase of natural killer cells in the peripheral blood. Reduction of T cell development in *Rag1* mutant rats was associated with a hypoplastic thymus that lacked follicular structures. Histological evaluation also revealed the near-complete absence of lymphocytes in spleen and lymph nodes in the immunodeficient *Rag1* mutant rat.

**Conclusion:**

The *Rag1* mutant rat will serve as an important model for transplantation studies. Furthermore, it may be used as a model for reconstitution experiments related to the immune system, particularly with respect to different populations of human lymphocytes, natural killer cells and autoimmune phenomena.

## Background

Several modern techniques facilitate the genetic manipulation of the rat genome *in vivo*. Aside from the pronuclear injection of recombinant DNA constructs, the integration of lentiviral vectors into the rat genome after injection into the perivitelline space and transposon-mediated insertions have enabled studies in transgenic rats [1-5]. Random integration of Sleeping Beauty and PiggyBac transposons caused gene inactivation at the integration sites [6,7]. Moreover, the cultivation of rat embryonic stem cells does allow gene targeting and functional deletion through homologous recombination (HR) *in vitro*[8-11]. However, due to the technical complexity of these procedures only the *p53* gene and the *Par-2* gene have been inactivated through HR in ES cells to date [12,13]. Alternative strategies for the functional inactivation of genes use the highly efficient Zinc-finger nuclease (ZFN) [14] and transcription activator-like effector nucleases (TALEN) technologies [15], both of which offer a rapid approach in gene targeting. Zinc-finger nucleases are hybrid proteins consisting of polymeric zinc-finger proteins fused to the endonuclease domain of the restriction enzyme *Fok*I. A pair of zinc-finger nucleases binds to two contiguous target sequences in each DNA strand separated by a 6 bp cleavage site. Subsequent dimerization of the *Fok*I domains causes DSB and initiate endogenous repair processes. Inaccurate repairs by NHEJ in response to DNA damage introduce deletions or insertions in the spacer region. The resulting frame shift leads to a premature stop codon and the translation of a truncated, inactive protein [16,17]. Therefore, ZFN have been used for the manipulation of human and rodent cells *in vitro*[18-20] as well as for gene targeting or for homology directed repair in zebra fish, mouse, rat and pig embryos *in vivo*[21-25]. In the rat, genes such as immunoglobulin heavy chain 6 (*Igh-6*), immunoglobulin heavy chain (gamma polypeptide) (*JH* locus), transgenic *eGFP*, *renin* (*Ren*), *interleukin 2 receptor gamma* (*Il2rg*), *ATP-binding cassette, sub-family B (MDR/TAP), member 1A (Mdr1a)* and *Rab38* have been successfully inactivated by ZFN mediated gene disruption in single cell embryos [26-30].

Here we report about generating a *Rag1* knockout by ZFN in LEW rats, with the intention of establishing an immunodeficient rat model on a defined genetic background.

*Rag1* as well as *Rag2* are highly conserved genes located in close vicinity in head to tail arrangement on chromosome 11p13 in humans, on chromosome 2qE2 in the mouse, and at chromosome 3q31 in the rat. In humans, *Rag1* mutations lead to severe combined immunodeficiency (SCID) due to B and T cell loss (T^-^B^-^SCID) or Omenn’s Syndrome (T^+^B^-^SCID) with a shortened life expectancy reviewed in [31-34]. A similar phenotype was described after gene targeting of *Rag1* in the mouse with an arrest of B and T precursor cells in an early developmental stage, no serum IgG and an elevated number of natural killer cells (NKC) [35-38].

The generated *Rag1* mutant rat on a defined genetic background can provide a useful tool for studies of the immune system, in transplantation studies, and in autoimmune diseases.

## Results

### Embryo manipulation and *Rag1* gene targeting

**Figure 1:**
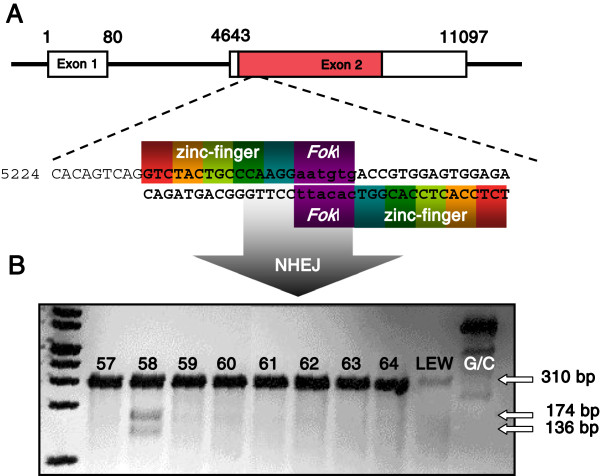
**Introduction of a frame shift mutation. A** The 11097 base pairs of genomic *Rag1* are separated in two exons. Of these only Exon 2 contains the complete coding sequence (red bar). *Rag1* specific zinc-finger nucleases recognizing 18 bp upstream and downstream of the 6 bp cutting site between position 5233 and 5267 introduced DSBs through the *Fok*I endonuclease domain and initiated NHEJ. **B***Cel*-I endonuclease digest of the 310 bp heteroduplex DNA derived from the hybridization of the DNA from LEW wild type rats and founder 58 into 174 bp and 136 bp fragments proofed the mutagenesis of one *Rag1* allele in founder 58. G/C: heteroduplex control DNA; LEW: LEW/Ztm wild type DNA.

A pair of zinc-finger nucleases consisting of five zinc-finger modules recognizing 15 bp at both sides of a 6 bp cut side located close to the 5’ end of Exon 2, were used to induce DSBs and NHEJ in the *Rag1* gene (Figure 1A). The injection of the *Rag1* specific ZFNs into 623 zygotes harvested from 35 hyperovulated donors resulted in 444 morphologically intact embryos transferred to 19 pseudopregnant (LEW x WKY) F1 females. From the 59 pups born, 49 survived the weaning period.

Surveyor mutation detection assay was performed to identify offspring carrying a *Rag1* mutation by cutting of heteroduplex DNA derived from hybridization of wild type LEW/Ztm DNA with founder DNA through the *Cel*-I endonuclease [39,40]. Analysis of the offspring revealed partial cleavage of the 310 bp hybrid DNA into 174 bp and 136 bp fragments in one female (denominated “founder 58”), giving a 1.7% rate of transgenesis of the *Rag1* gene (Figure 1B).

### *Rag1* frame shift mutation

**Figure 2:**
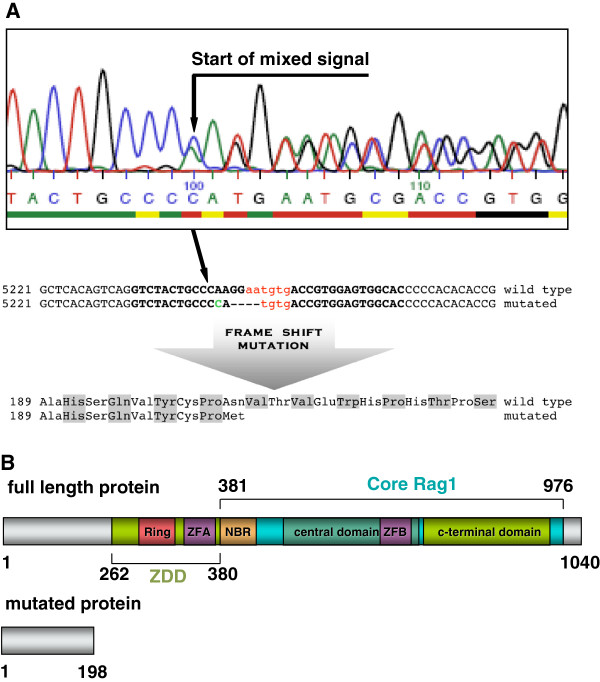
**Analysis of the mutant *Rag1 *allele. A** Heterozygosity at the genomic *Rag1* locus of founder 58 was confirmed by sequencing. The start of the mixed signal indicated the *Rag1* mutation identified as A to C exchange (green upper case) plus 4 bp deletion at the ZFN cut site (red lower case) and the ZFN binding regions (black upper case). The introduced frame shift caused a premature stop codon leading to a truncation of the *Rag1* protein to a 198 aa N-terminal residue. **B** Full length *Rag1* protein consists the functional important zinc-binding dimerization domain (ZDD) containing the RING finger (RING) and the zinc finger A (ZFA) as well as the c-terminal core domain with the nonamer-binding region (NBR) and the zinc finger B (ZFB). Mutated *Rag1* lost all domains essential for *Rag1* function in V(D)J recombination.

**Figure 3:**
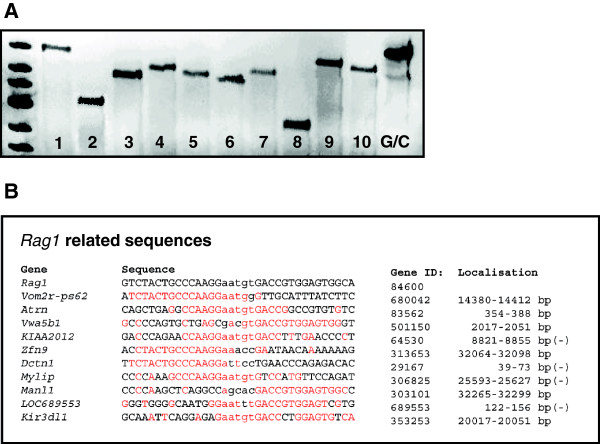
**Off-target mutation analysis. A** Cel-I digest of heteroduplex DNA revealed no additional off-target mutations at the 10 loci with highest homology to *Rag1*; 1: Vom2r-ps62; 2: Atrn; 3: Vwa5b1; 4: KIAA2012; 5: Zfn9; 6: Dctn1; 7: Mylip; 8: Manl1; 9: LOC689553; 10: Kir3d1l; G/C: control heteroduplex DNA. **B** Genes, gene IDs, sequence homologies and localization of *Rag1* related sequences to exclude off-target mutations; upper case: ZFN binding sites; lower case: ZFN cut site; homolog base pairs in red; (-): Gene located on the minus strand.

Sequencing of exon 2 of the *Rag1* gene from “founder 58” verified an A to C base exchange at position 5244 combined with a 4 bp deletion at position 5246 – 5249 while mutations were absent on the second allele (Figure 2A). The 4 bp deletion in the *Rag1* gene of “founder 58” caused a frame shift mutation and generated a premature stop codon at position 667 of the *Rag1* mRNA, thereby encoding a truncated 198 aa protein. This shortened protein was homologous to the N-terminal part of wild type *Rag1* but lost the zinc-binding dimerization domain (ZDD) and the c-terminal Core domain of the 1040 aa full length protein [38] (Figure 2B). No off-target mutations were seen in the 10 loci with the highest sequence homology to the zinc-finger binding and cut site in the *Rag1* gene by the SURVEYOR mutation detection assay in “founder 58” (Figure 3).

### Germ line transmission and genotyping

**Figure 4:**
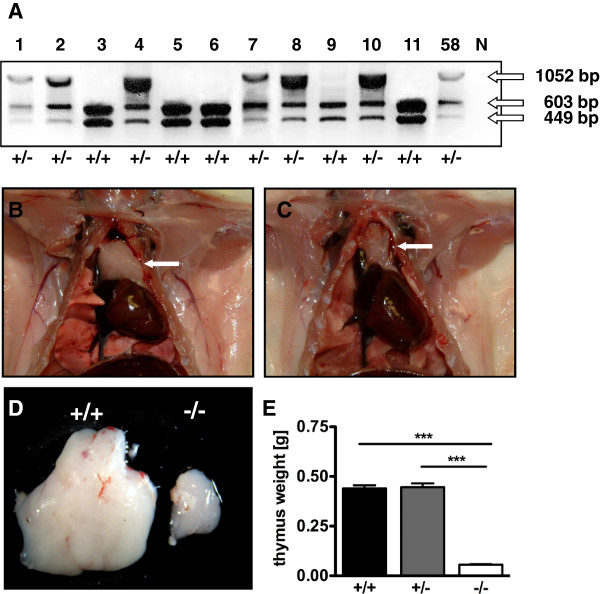
**Genotyping and macroscopical examination. A** Genotyping of the offspring derived from founder 58 using PCR amplification and *Sty*I-HF digest. Half of the 1052 bp PCR products from heterozygous animal are digested into 603 bp and 449 bp fragments, while the *Rag1* PCR product from wild type offspring was digested completely; N: negative control. 58: PCR product from founder 58 as positive control. **B** Normal thymus of wild type animal of the strain LEW-*Rag1*em1/Ztm-*Ragl*^*eml*Ztm^. **C** Hypoplastic thymus in a *Rag1* mutant rat. **D** Comparison of the thymus sizes of wild type and *Rag1* mutant rats. **E** Comparison of thymus weigths, *** : P < 0.0001.

Subsequently, the heterozygous “founder 58” was mated to a wild type LEW male to evaluate germ line transmission and to establish the novel *Rag1* deficient LEW/Ztm-*Rag1*^*em1*Ztm^ coisogenic strain. To distinguish between wild type and heterozygous pups, we used the loss of the recognition site of the restriction endonuclease *Sty*I caused by the 4 bp deletion in Exon 2 of *Rag1*. Therefore, genotyping was performed by PCR amplification of a 1052 bp sequence of *Rag1*. While the PCR products derived from wild type allele were cleaved into 603 bp and 449 bp fragments by *Sty*I, mutated *Rag1* sections remained uncut. Six out of 11 offspring were heterozygous, demonstrating germ line competence and confirming the heterozygosity of “founder 58” (Figure 4A). Offspring carrying the mutation was used for further breeding. Heterozygous LEW/Ztm-*Rag1*^*em1*Ztm^ rats showed normal reproduction with litter sizes ranging from 6 to 12. The mutation was inherited in the expected Mendelian ratio.

### Macroscopical and histological evaluation

**Figure 5:**
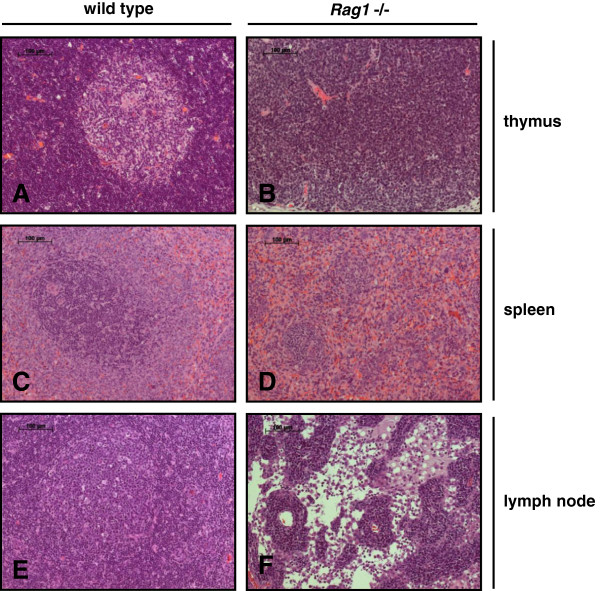
**Histological evaluation of lymphatic organs of wild type and *Rag1 *mutant rats. A** and **B** thymus; **C** and **D** spleen; **E** and **F** lymph nodes. H/E staining.

Phenotypical characterization revealed no ontogenetic disorders despite of underdeveloped thymi of the *Rag1* mutant rats (Figure 4B and C). The mean thymus weight was significantly reduced from 0.44 g (SD ±0,04) in wild type and 0.45 g (SD ±0,05) in heterozygous animals to 0.06 g (SD ±0,01) in homozygous *Rag1* mutant rats (Figure 4D and E). The reduced size of the thymus of *Rag1* mutant rats was associated with a complete loss of the follicular structure showing the absence of T lymphocytes (Figure 5B). Histological evaluation of the spleen demonstrated the differentiation in red and white pulp containing B cells in the lymphatic follicles and T lymphocytes in the periarteriolar lymphoid sheaths (PALS) in wild type animals (Figure 5C). Besides the central artery the follicles of the white pulp are reduced and the PALS were lost in rats with partial *Rag1* inactivation (Figure 5D). In wild type lymph nodes the cortex was characterized through trabecular structures separating follicles where B cell differentiation occurred in the germinal centers (Figure 5E). The lymph nodes of homozygous LEW/Ztm-*Rag1*^*em1*Ztm^ rats appeared devoid of T and B lymphocytes and follicular structures, in which physiological B cell differentiation would occur, were absent (Figure 5F). No further macroscopic differences in organ size and structures were found in homozygous LEW/Ztm-*Rag1*^*em1*Ztm^ rats, and careful histological evaluation revealed no malformation of organs outside of the lymphatic system.

### Disruption of wild type *Rag1*

**Figure 6:**
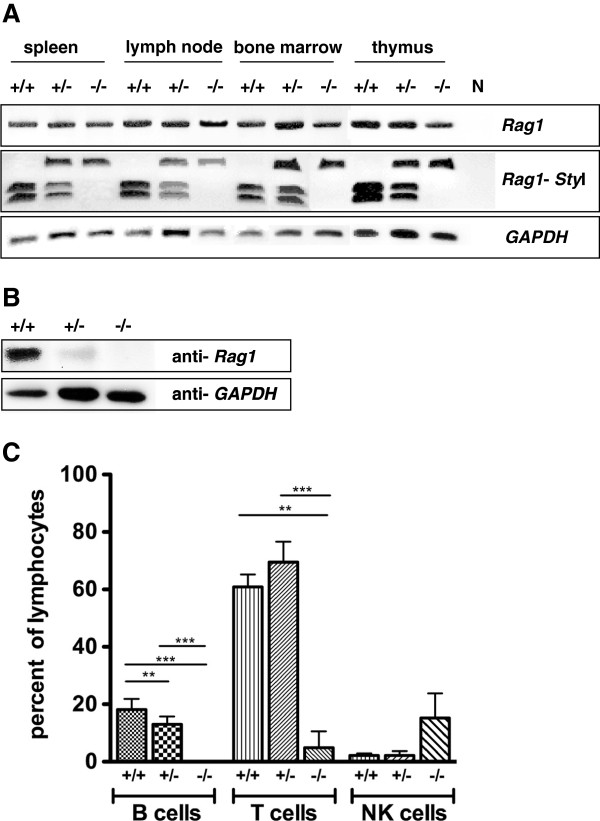
**Analysis of *Rag1 *expression in lymphatic organs.** RT-PCR of wild type, heterozygous and *Rag1* mutant rats: The *Rag1* PCR product (upper panel) was digested with *Sty*I-HF to distinguish between translation of wild type and mutated *Rag1* (mid panel). *GAPDH* used as housekeeping gene (lower panel). **B** Western Blot analysis: Translation of *Rag1* in the thymus from wild type, heterozygous and *Rag1* mutant rats compared to *GAPDH* as housekeeping protein. **C** Comparison of NK cells, B and T cell ratio in the peripheral blood of wild type, heterozygous and *Rag1* mutated rats determined by FACS analysis. *** P < 0.0001; ** P < 0.005.

Molecular characterization by RT-PCR showed a shift from the expression of wild type *Rag1* mRNA in the lymphatic organs of wild type rats to the exclusive expression of mutated *Rag1* in knockout rats. In heterozygous animals wild type and mutated *Rag1* were transcribed equally (Figure 6A). Western Blot analysis confirmed the loss of full length *Rag1* protein in the thymus of homozygous mutant rats, while heterozygous animals exhibited a significant reduction of the *Rag1* protein concentration as compared to the strong translation of *Rag1* in wild type rats (Figure 6B).

### Lymphocyte depletion in *Rag1* mutant rats

The quantity of B and T lymphocytes, as well as NKC was determined by FACS analysis. The fraction of B cells in peripheral blood was almost identical in wild type and heterozygous animals with a mean of 18,1% (SD ± 3,4%) and 13% (SD ± 2,5%). B cells were absent in *Rag1* homozygous mutant rats (Figure 6C; 7A-C). Further, the ratio of T cells was almost the same in wild type and heterozygous rats with 60,9% (SD ± 4,1%) and 69,5% (SD ± 6,9%), however in rats with deficiency of full length *Rag1* T cells were significantly reduced with 4,6 (SD± 5,7%) (Figure 6C; 7A-C). Inverse ratios were found when counting NKC. While in wild type and heterozygous rats only 2,2% (SD ± 0,7%) respectively 2,2% (SD ± 1,2%) of NKC could be detected, the peripheral blood of *Rag1* mutant rats contained 15,3 ± 7,5% NKC suggesting a compensatory increase of natural killer cells (Figure 6Cx; 7A-C). Further analysis revealed that the T cell population isolated from the peripheral blood of rats with homozygous *Rag1* frame shift mutation consisted of mature CD4^+^/CD3^+^/TCRαβ^+^ and CD8^+^/CD3^+^/TCRαβ^+^ thymocytes (Figure 8).

**Figure 7:**
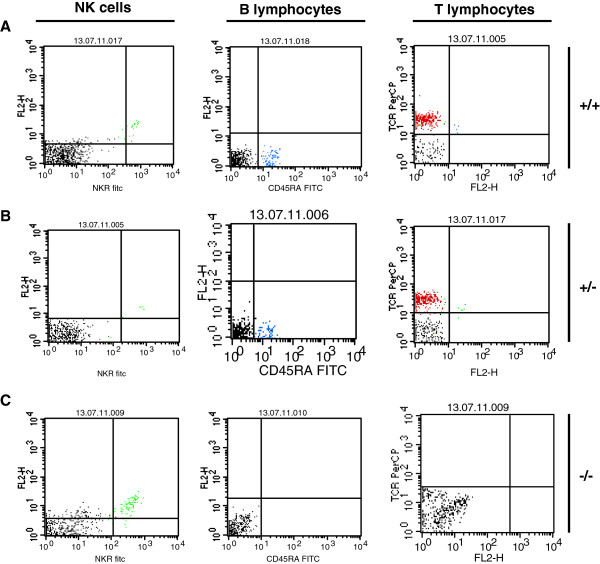
**FACS analysis of NKC, B and T lymphocytes in the blood: A wild type, B heterozygous and C *Rag1 *mutant rat; blue: CD161 positive NKC; green: CD45RA positive B cells; red: T cell receptor positive lymphocytes.**

**Figure 8:**
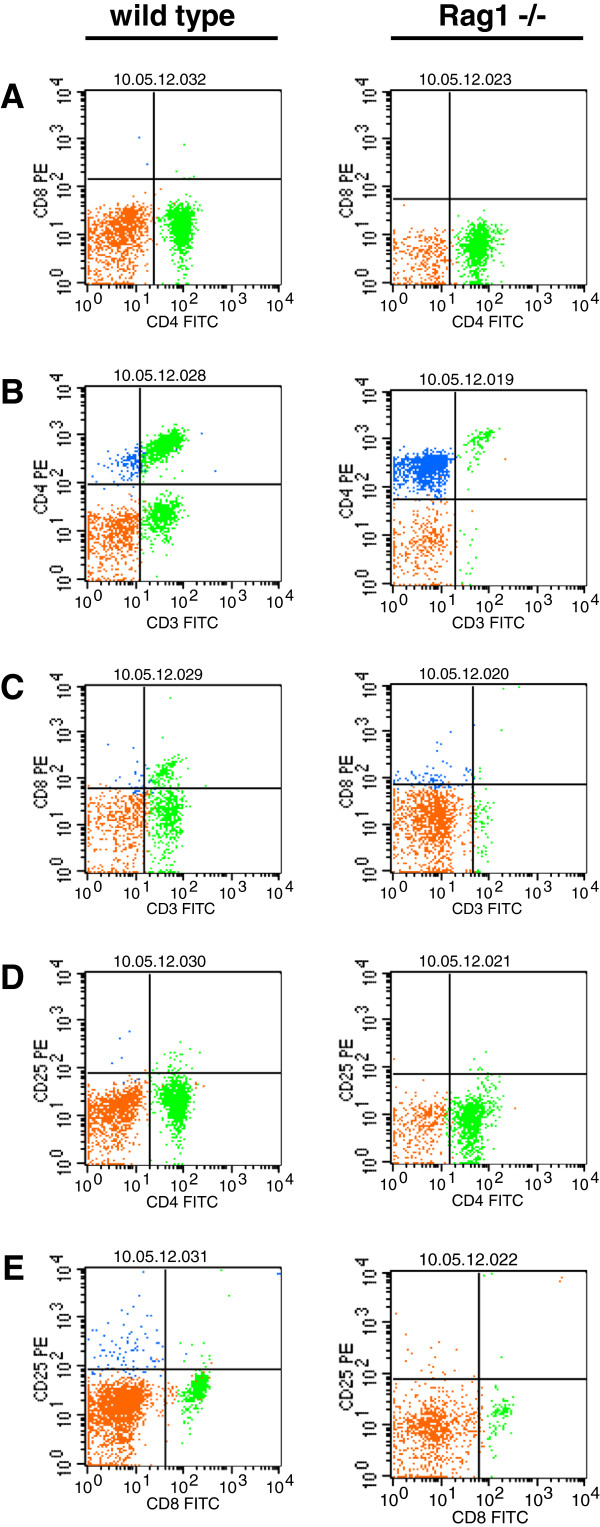
**Mature CD4 or CD8 positive T lymphocytes in the peripheral blood of *Rag1 *mutant rats. A** Absence of CD4^+^/CD8^+^ lymphocytes in wild type and *Rag1* mutant rats. **B** and **C** Reduction of CD4^+^/CD3^+^ and CD8^+^/CD3^+^ T cells. **D** and **E** While there are no differences in the population of CD25^+^/CD4^+^ T cells the number of CD25^+^/CD8^+^ lymphocytes was reduced. orange: autofluorescent cells; green: FITC positive lymphocytes; blue: PE positive lymphocytes.

## Discussion

### Low efficiency of *Rag1* specific ZFNs in LEW/Ztm rats

The most striking advantage of the ZFN technology is the capability to manipulate embryos of any species. Nevertheless, the efficiency of the genetic manipulation may be modulated by strain variations and species differences, as well as locus specific chromatin structure and technical setup. In LEW/Ztm 71,3% of the injected embryos were still viable and resulted in 13,3% live births. However, only 1,7% carried the expected *Rag1* mutation. In contrast to this the workgroup at the SAGE Lab, which used the identical ZFN mRNAs for the disruption of *Rag1* in SD rats achieved a ratio of 20% of *Rag1* mutated rats (5 out of 24 pups born, personal communication).

The efficiency of 3% (1 out of 31 born animals carrying the mutation) seen in the targeting of the *Renin* gene in inbred SS (salt-sensitive) rats [30] was at a similar low level as the ablation of *Rag1* in the LEW/Ztm background. Guerts and coworkers found survival rates comparable to the LEW rat with 56% to 91% after microinjection of ZFN encoding plasmids or ZFN mRNA in the SD background. The ratio of live-born pups born (13 - 20%) also correlates with our results, however, the efficiency of the manipulation was much higher in the SD rats up to 75% mutants [27] instead of 1,7% on the LEW background. Nevertheless, Mashimo and colleagues detected high average ratios of born and mutated animals with 24,3% and 24,1% after micromanipulation of zygotes from inbred F344/Stm or TM/Kyo strains to inactivate the *IL2rg* gene [28]. However, comparing the efficiency of the *Rag1* mutation in outbred SD and inbred LEW suggests that the different genetic backgrounds are an important determinant in the ratio of transgenesis, even though it also depends on the accessibility of the gene locus due to the chromatin structure [41].

Further, the conditions of the technical engineering determined the success of the ZFN mediated gene targeting. Guerts and coworkers showed that the stepwise increase of the concentration of the injected mRNAs from 0.4 μg/μl to 2μg/μl and then to 10 μg/μl correlates with reduced ratios of born embryos. Among these the number of mutant offspring increased with the injection of higher mRNA concentrations in SD rats [27]. Therefore, the functionality of the individual pair of ZFNs and the technical setup of the experiment in combination with the stress sensitive and delicate LEW embryos might be responsible for the low outcome of *Rag1* mutated rats in this study [42]. Future comparison of identical ZFN in several rat inbred strains will be necessary to validate strain dependent variations of ZFN mediated manipulation efficiencies.

### High fidelity of the *Rag1* ZFN

The introduction of genetic modifications in the mammalian genome harbors the risk of causing unwanted, random or off-target mutation. It is well known that the Cre recombinase widely used for the conditional deletion of genomic sequences recognizes cryptic “pseudo-loxP” sites, initiates chromosomal rearrangements and infertility in mice [43,44]. Despite the high binding specificity of ZFN to their specific target sequence additional off-target mutations at homologous loci must be excluded. Neither in our LEW-*Rag1* mutant rat, nor in any other published knockout rat model created by ZFN-mediated genome modification, off-target mutations have been detected so far, thereby demonstrating the high fidelity of ZFN binding and activity [21,26-30].

### *Leakiness of* LEW/Ztm*-Rag1*^*em1*Ztm^

The phenotype of the LEW-*Rag1* mutant rat was characterized by a substantial decrease of *Rag1* translation in heterozygous animals and by a complete loss of the *Rag1* protein in the homozygous mutant rats suggesting a biallelic expression of *Rag1* in wild type rats. The absence of dose dependent effects in the heterozygous animals emphasized that the translation of *Rag1* from only one allele was sufficient for a functional immune system while homozygous *Rag1* mutation was associated with a reduction of T cells, complete ablation of B cells and an elevation of NK cell number in peripheral blood demonstrated by FACS analysis. The repression of lymphocyte development due to the lack of V(D)J recombination was associated with a hypoplastic thymus and the loss of follicular structures associated with lymphocyte maturation in thymus, spleen and lymph nodes. The underdevelopment of the thymus in the LEW-*Rag1* mutant rat correlates with the reduced size of the thymus in the *Rag1* knockout mouse, where the substitution of a central part of the coding sequence in exon 2 through a neomycin resistance cassette led to the complete functional inactivation of *Rag1*[37]. In contrast to the non-leaky *Rag1* knockout mouse we found an average of 4,6 % T cell receptor positive thymocytes while no B cells were found in the LEW-*Rag1* mutant rat. Furthermore, in the SD-*Rag1* mutant rat [45] nearly 11% CD3 positive T cells and some CD45RA expressing B cells were detected. *Rag1* deficiency excludes lymphocyte maturation as shown in the *Rag1* knockout mouse. T cell precursors mature only up to the double negative CD4^-^CD8^-^IL2R-α positive stage in the absence of murine *Rag1*[35,37]. Therefore, the origin of mature T lymphocytes in the *Rag1* knockout rat is still unknown as extrathymic thymocyte maturation, such as intestinal intraepithelial TCRγδ^+^ T lymphocytes (IEL) in BB rats, CD2^+^CD7^+^ preTCRα^+^ T cells in the adult liver or CD2^+^CD7^+^CD3^-^ T cells in the human small intestine, always require the expression of functional *Rag1*[46-48]. A similar frame shift mutations like in the LEW/Ztm-*Rag1*^*em1*Ztm^ were observed in the human *Rag1* gene in two patients with Omenn’s syndrome carrying an adenosine deletion at position 877 of the *Rag1* cDNA also introducing a premature stop codon in front of the nonamer binding region (NBR) [49,50]. Santagata and colleagues showed that in response to the frame shift mutation truncated *Rag1* protein had been translated from an internal methionine without the N-terminal BIIA nuclear localization signal. Like in LEW/Ztm-*Rag1*^*em1*Ztm^ homozygous rats, in these patients differentiation of mature, activated but oligoclonal T cells with impaired function took place due to cytoplasmic localization of the truncated *Rag1* and reduced recombinational activity [49,50]. In the future cloning of the LEW/Ztm-*Rag1*^*em1*Ztm^ allele followed by *in vivo* translation and recombination should demonstrate the translation, cellular localization and functionality of the mutated *Rag1* protein in the LEW/Ztm rats.

### Functional compensatory NK cell increase

The elevated NK cell number in the peripheral blood of LEW/Ztm-*Rag1*^*em1*Ztm^ rats correlates with the observation of an increase in NKC population in spleen, lymph nodes, lung, and liver of *Rag1* knockout mice [36]. Grundy and Sentman speculate that the NKCs substitute T lymphocytes by migration along chemokine gradients into these organs. The increase in NK cell concentration in the blood of LEW/Ztm-*Rag1*^*em1*Ztm^ mutant rats was also associated with a reduction of thymocytes, but the molecular pathways involved in this compensatory process are still unknown.

Further characterization of the LEW/Ztm-*Rag1*^*em1*Ztm^ strain as well as xenotransplantation experiments of cells and tissues will be performed to establish the *Rag1* mutant rat as a innovative model for studies in autoimmune diseases, transplantation approaches and carcinogenesis as well as cancer therapy.

## Methods

### Animals

All rats were bred and maintained at the Central Animal Facility of the Hannover Medical School, Carl-Neuberg-Strasse 1, 30625 Hannover, Germany (subline code: Ztm: http://www.mh-hannover.de/einrichtungen/tierlabor). All handling of animals has been conducted in accordance with German law for animal protection and with the European Communities Council Directive 2010/63/EU for the protection of animals used for experimental purposes. All experiments have been approved by the Local Institutional Animal Care and Research Advisory committee and permitted by the local government (Lower Saxony State Office for Consumer Protection, Food Safety, and Animal Welfare Service – LAVES; Az.10/0051).

### Husbandry

LEW, LEW/Ztm-*Rag1*^*em1*Ztm^, and (LEW x WKY)F1 rats were maintained under standardized conditions at a temperature of 22 ± 2°C, relative humidity of 55±5%, and artificial light for 14h. Commercial softwood granulate bedding was sterilized (Lignocel, Altromin; Lage, Germany). They received an autoclaved commercial pelleted diet (Altromin 1314) and water *ad libitum*. The rats were kept as pairs or in sibling groups. Microbiological status was monitored according to FELASA recommendations [51]. The rats were positive for parvovirus and apathogenic protozoa.

### Zinc-finger nucleases

*Rag1* specific ZFN consisting of 5 zinc-finger modules fused to the endonuclease domain of the restriction endonuclease *Fok*I were designed and functionality evaluated by Sigma Advanced Genetic Engineering Labs using the CompoZr^®^ ZFN technology. The forward ZFN recognized the DNA sequence GTCTACTGCCCAAGG between positions 5233-5247, while the reverse ZFN detected the DNA segment GACCGTGGAGTGGCA located at 5253-5267 in Exon 2 of genomic *Rag1* (*Rattus norvegicus* strain BN/SsNHsdMCW chromosome 3, Rnor_5.0: NCBI Reference Sequence: NC_005102.3). The ZFN binding sites were separated by the 6 bp cut site. ZFN mRNAs were translated *in vitro* using the MessageMax T7 ARCA-Capped Message Transcription Kit (#MMA60710, Epicentre Biotechnologies, Madison, WI) and the Poly (A) Polymerase tailing kit (#PAP5104H, Epicentre Biotchnologies, Madison WI) following the manufacturer’s recommendations. mRNA quantity was measured using the Nanodrop spectrophotometer (Peqlab, Erlangen, Germany) and sufficient quality was confirmed by agarose gel electrophoresis.

### Embryo collection and manipulation

LEW/Ztm female were hyperovulated as described earlier [52]. Equal amounts of both ZFN mRNAs were mixed and diluted with injection buffer (1mM Tris-HCl, pH 7.4; 0.25 mM EDTA) to a final concentration of 5 ng/μl [21]. Estrous in LEW/Ztm was induced by injection of 50μg LHRH (L 4513, Sigma-Aldrich, Seelze, Germany) 4 days prior to mating. Zygotes were harvested on day 0.5 of pregnancy from the ampullae of oviducts in M2 medium (M7176, Sigma-Aldrich, Seelze, Germany) containing 2.2 mg/ml hyaluronidase (9978.1, Carl Roth, Karlsruhe, Germany) to detach the cumulus cells. Cumulus cell free zygotes were washed several times in M2 and than transferred to modified M16 medium supplemented with 1% non-essential amino acids (M7145, Sigma-Aldrich, Seelze, Germany) and covered with mineral oil (Light Mineral Oil EmbryoMax^®^, Millipore, Schwalbach, Germany). ZFN mRNAs were microinjected with glas capillaries (inner diameter of 1.2 μm, Pronucleus PI-1.2, BioMedical Instruments, Zöllnitz, Germany) into the cytoplasm of the zygotes with an injection pressure of 120 hPA (Ph: 80 hPA) using an Axiovert 135 microscope (Zeiss, Göttingen, Germany) with TransferMan NK2 and FemtoJet (Eppendorf, Hamburg, Germany). Injected zygotes were incubated over night in an incubator at 37°C, 5% CO_2_ and 95% humidity. Morphologically intact one-cell and two-cell embryos were transferred the next day into the oviducts of day 0.5 pseudopregnant (LEW x WKY) F1 rats [42].

### Mutation detection

*Rag1* from the putative founder animals and from wild type LEW rats was amplified with the Optimase^®^ Polymerase (Transgenomic, Glasgow, UK) and the primer pair Cel-I forward 5’-CTCATTGCCAGAGTTTTCCG-‘3 and Cel-I reverse 5’-TGCTGACCCTAGCCTGAGTT-‘3 using the following PCR protocol: 95°C for 5 min, 34 cycles: 95°C for 30 sec, 60°C for 30 sec, 72°C for 1 min); 72°C for 5 min, 4°C ∞. The resulting 310 bp fragments covering the ZFN cut site was hybridized and digested with the SURVEYOR^®^ Mutation Detection Assay (Transgenomic, Glasgow, UK) following the supplier’s instruction. Cel-I digestion of heteroduplex DNA derived from *Rag1* mutated animals resulted in cleavage of the 310 bp PCR product into 174 bp and 136 bp fragments. *Rag1* mutation was confirmed by sequencing of genomic DNA and PCR products with the ABI 310 Genetic Analyzer (Applied Biosystems, Darmstadt, Germany) using the BigDye^®^ Terminator v1.1 Cycle Sequencing Kit (Applied Biosystems, Darmstadt, Germany).

Performing blast search 10 genomic loci showing strong homology to the ZFN binding and cut site of *Rag1* were identified. Off-target mutations at these loci were excluded by PCR amplification at an annealing temperature of 58°C followed by Cel-I digest using the SURVEYOR^®^ Mutation Detection Assay (Transgenomic, Glasgow, UK) (Oligonucleotides see Additional file 1.

### Genotyping

The genotype of LEW/Ztm-*Rag1*^*em1*Ztm^ rats was determined by PCR amplification of a 1052 bp fragment of *Rag1* with the primer pair *Rag1*for1: 5’-AGGTAGCTTCGCCAAA.

ATGG-‘3 and *Rag1*rev1: 5’-TCAGAAAGGACTTGACCGGA-‘3 using the HotStarTaq Master Mix Kit (Qiagen, Hilden, Germany) following the manufacturer’s recommendations. PCR were performed in a volume of 50 μl according to the following protocol: 95°C for 15 min; 40 cycles: 95°C for 15 sec, 60°C for 30 sec, 72°C for 1 min; 72°C for 5 min; 4°C ∞. After reaction clean up with the Qiaquick Gel Extraction Kit (Qiagen, Hilden, Germany) the PCR products containing the *Rag1* mutation were cleaved by *Sty*I-HF (NEB, Frankfurt, Germany) into 603 bp and 449 bp, while the wild type *Rag1* fragments remained unchanged.

### RNA and RT-PCR

Total RNA was isolated from spleen, thymus, bone marrow and lymph nodes (mesenteric and cervical) from wild type LEW/Ztm rats, heterozygous and homozygous *Rag1* mutant rats (n=10) using the RNeasy Mini Kit (Qiagen, Hilden, Germany) after homogenization of the tissues with the UP100H ultrasonic device (Hielscher, Teltow, Germany) and Qiashredder (Qiagen, Hilden, Germany). RNA concentrations were determined with the Nanodrop spectrophotometer (Peqlab, Erlangen, Germany). 2 μg of pooled RNA were transcribed to cDNA with the Omniscript RT Kit (Qiagen, Hilden, Germany), Random Hexamers (Fermentas, St. Leon-Rot, Germany) and Protector Rnase Inhibitor (Roche, Mannheim, Germany). Rat GAPDH was amplified with the following primers 5’-AGGGCTGCCTTCTCTTGTGAC-‘3 and 5’-CCGTGGGTAGAGTCATAC TGG-‘3 with an annealing temperature of 57°C for 25 cycles using the HotStarTaq Master Mix Kit (Qiagen, Hilden, Germany). Expression of wild type and mutated *Rag1* was determined using the genotyping protocol.

### Western blot

Whole cell protein extracts were isolated from spleen, thymus, bone marrow and lymph nodes (mesenteric and cervical) from wild type, heterozygous, and *Rag1* deficient rats (n=10) with NP40 buffer supplemented with Complete Mini Protease Inhibitor Cocktail Tablets (Roche, Mannheim, Germany), sodium orthovanadate (NEB, Frankfurt, Germany), PMSF (Sigma-Aldrich, Seelze, Germany) and DTT (Sigma-Aldrich, Seelze, Germany). Bradford Assay served to estimate the protein concentration. 50 μg of protein supplemented with 5x LSB loading dye was separated in a 10% gel by SDS-PAGE and transferred to Optitran BA-S85 membrane by semi-dry blotting. After blocking for 1 hour at room temperature with blocking solution (5% dry milk (Carl Roth, Karlsruhe, Germany) and 2,5% BSA, fraction V (Sigma-Aldrich, Seelze, Germany) in 3% TBS-Tween, pH 7,4) the membrane was incubated with the first antibody diluted in blocking solution at 4°C over night. Incubation with the secondary, HRP-conjugated antibodies was done for 1 hour at room temperature in 3% TBS-Tween, pH 7,4. Roti-Lumin^®^ was used as chemoluminescence substrate. For detection of the *Rag1* protein a 1:300 dilution of the RAG-1 antibody (sc-5599, SCBT, LaJolla, CA) was sufficient followed by incubation with a 1:2500 dilution of a donkey anti rabbit secondary antibody (ab16284, Abcam, Cambridge, UK). GAPDH expression was demonstrated with a 1:300 dilution of the anti-GAPDH antibody (OBT1636, AbD Serotec, Düsseldorf, Germany) in combination with a 1:5000 dilution of the rabbit anti mouse antibody (STAR13B, AbD Serotec, Düsseldorf, Germany).

### Histology and thymus weight

Tissues were fixed in 4% formaldehyde solution for 3 days, paraffin-embedded and cut into 4 μm slices before staining with hematoxylin and eosin. Microphotographs were taken using a Zeiss AxioCam MRc camera and analyzed histologically. Thymus weight was measured using a LA230S scale (Sartorius, Göttingen, Germany). One-way analysis of variance (ANOVA) and Bonferroni's Multiple Comparison Test were performed using Graph Pad Prizm Software (Version 5.0a) to determine statistical significances with a threshold of P < 0.05.

### FACS analysis

Blood was harvested from wild type, heterozygous and *Rag1* deficient rats (n=10), and lymphocytes were isolated following standard procedures. Cells were incubated for 30 min at 4°C with a 1:10 dilution of the anti-CD45RA FITC (sc-53048, SCBT, LaJolla, CA) antibody to detect B cells, while the number of T cells and natural killer cells were determined by double staining with 1:10 dilutions of the PerCP mouse anti-rat αβ T-cell receptor (BD Pharmingen, Heidelberg, Germany) and the mouse anti rat CD161:FITC antibody (AbD Serotec, Düsseldorf, Germany). Double stainings were performed using mouse anti rat CD3:FITC/CD4:RPE dual color reagent (DC041), mouse anti rat CD3:FITC/CD8:RPE (DC042) and mouse anti rat CD4/CD25 (DC040) (Abd Sertec). For CD4/CD8 double staining the antibodies mouse anti rat CD4-Fitc (554843, BD Pharmingen) and mouse anti rat CD8A-PE (554857, BD Pharmingen) were used. Stained cells were measured together with unstained cells and isotype controls stained with 1:50 dilutions of the PerCP Mouse IgG1 κ isotype control antibody (BD Pharmingen, Heidelberg, Germany) or the Mouse IgG (FITC) antibody (ab37356, Abcam, Glasgow, UK) with the FACSCalibur flow cytometer (BD Pharmingen, Heidelberg, Germany). ARCSIN transformation, One-way analysis of variance (ANOVA) and Tukey’s Multiple Comparison Test were performed using Graph Pad Prizm Software (Version 5.0a) to determine statistical significances with a threshold of P < 0.05.

## Abbreviations

ZFN: Zinc-finger nucleases; DSB: DNA double strand breaks; NHEJ: Non-homologous end joining; TALEN: Transcription activator-like effector nuclease; SCID: Severe combined immunodeficiency; NKC: Natural killer cells; ZDD: Zinc-binding dimerization domain; NBR: Nonamer binding region; PALS: Periarteriolar lymphoid sheaths.

## Competing interests

EJW and XC are full-time of employees of SAGE Labs of Sigma-Aldrich Corporation. NHZ, SG, DW, MD and HJH declare that they have no competing interests.

## Authors’ contributions

NHZ carried out the microinjection, genotyping, molecular biological analysis and drafted the manuscript. SG performed the histopathological examinations. DW was responsible for the FACS analysis. EJW and XC validated the *Rag1* ZFNs and provided technical advice. MD and HJH participated in the study design. All authors read and approved the final manuscript.

## Supplementary Material

Additional file 1 **Off-target gene loci:*RAG1 ZFN mutagenesis.*** Click here for file
