# Supplementary figures and images for: Zinc-finger nuclease mediated disruption of Rag1 in the LEW/Ztm rat
Source: BMC Immunol. 2012 Nov 8;13:60. doi: 10.1186/1471-2172-13-60 (PMC3522011; doi:10.1186/1471-2172-13-60)

## Slide 1
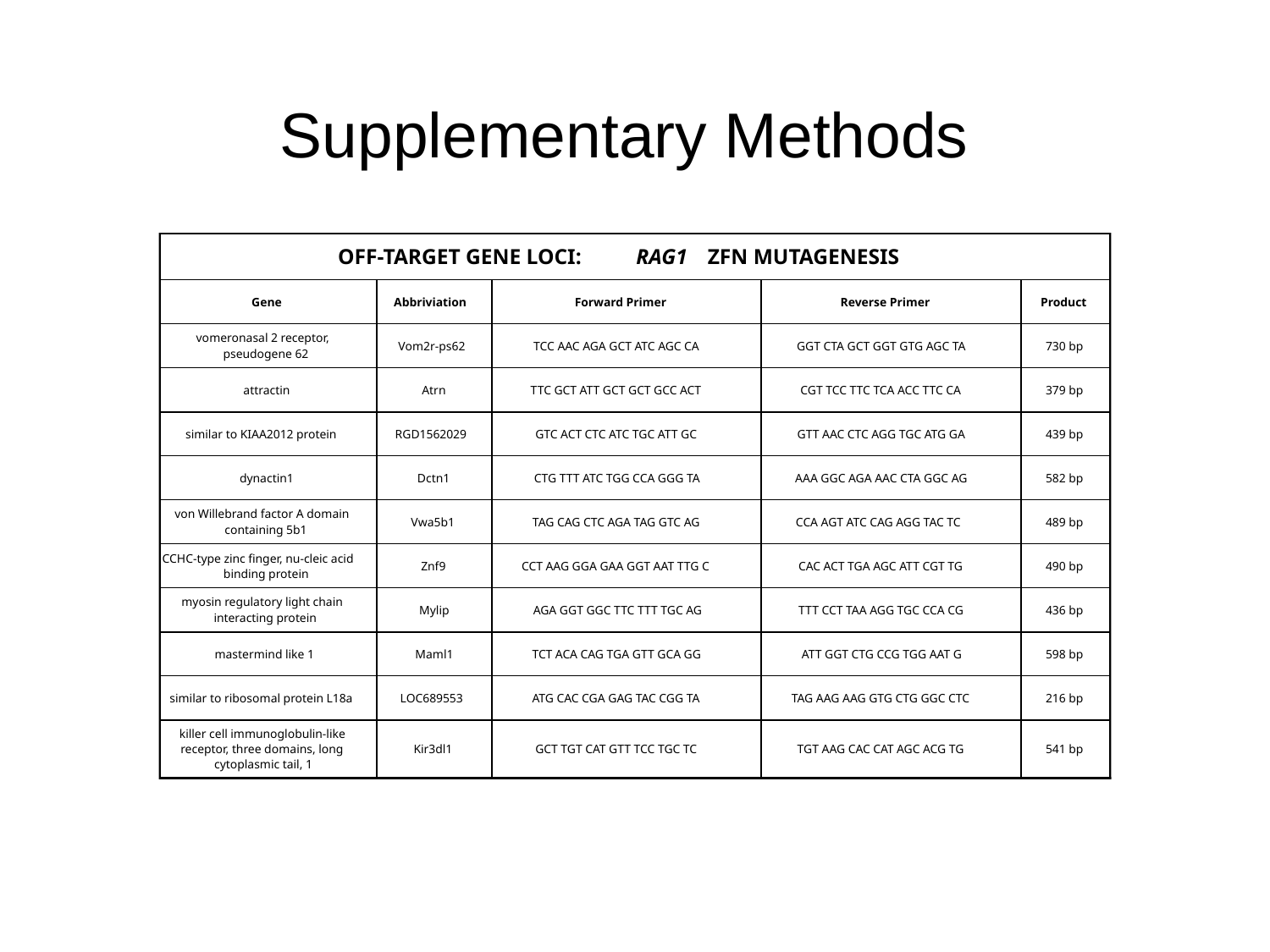

# Supplementary Methods

Supplement: Additional file 1 — Off-target gene loci:RAG1 ZFN mutagenesis. [file 1471-2172-13-60-S1.ppt]
